# Polydatin ameliorates ovalbumin‐induced asthma in a rat model through NCOA4‐mediated ferroautophagy and ferroptosis pathway

**DOI:** 10.1002/2211-5463.70090

**Published:** 2025-07-16

**Authors:** Wei Li, Yuwei Tang, Wenkang Liu, Fang Fang, Jiepeng Wang, Chaoyi Fang

**Affiliations:** ^1^ College of Traditional Chinese Medicine Hebei University of Chinese Medicine Shijiazhuang China; ^2^ Hebei Key Laboratory of Integrated Chinese and Western Medicine for Lung Disease Research Shijiazhuang China

**Keywords:** asthma, ferroautophagy, ferroptosis, polydatin

## Abstract

Asthma is one of the most prevalent chronic diseases worldwide. In this study, we aimed to explore whether polydatin can achieve therapeutic effects in experimental asthma in a rat model by suppressing ferroptosis and its potential mechanism of inhibiting ferroptosis. We established a rat asthma model, and five experimental groups were established: the control group, model group, polydatin group, 3‐MA group, and Fer‐1 group. We compared general conditions, behavioral changes, Fe^3+^deposition, pathological changes, pulmonary function, serum IgE levels, ferroautophagy‐related genes, and ferroptosis‐related genes expression among the groups. Following the polydatin intervention, the mental state of the rats stabilized, their fur condition improved, and both food intake and body weight increased. The incubation period of asthma lengthened, and they sneezed and scratched less frequently. Additionally, polydatin reduced serum IgE levels and Fe^3+^ deposition, enhanced lung function and pathological alterations, and also downregulated the expression of nuclear receptor coactivator 4 (NCOA4), Bcl‐2 homologous domain protein (Beclin1), Fe^2+^, malondialdehyde (MDA), and 4‐hydroxynonenal (4‐HNE) in lung tissue. Levels of ferritin heavy chain 1 (FTH1), ubiquitin‐binding protein p62 (P62), glutathione (GSH), glutathione peroxidase 4 (GPX4), and solute carrier family 7 member 11 (SLC7A11) were all upregulated. In conclusion, in this rat model, polydatin was capable of reducing Fe^2+^ overload by inhibiting the NCOA4‐mediated ferroautophagy. This, in turn, inhibited ferroptosis in the lung tissues, thereby alleviating asthma symptoms. Further studies, including clinical trials, are required to validate this result.

## Graphical abstract

In this research, a rat model of asthma was created using OVA, and polydatin served as an intervention. By inhibiting ferroautophagy mediated by NCOA4 and averting ferroptosis, polydatin has been demonstrated to reduce asthma. This work presents new ideas for investigating the mechanism of polydatin's ability to alleviate asthma, in addition to experimental support and theoretical underpinnings for this claim.

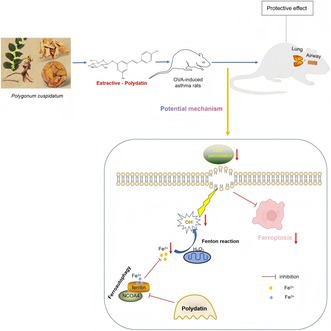

**Abbreviations**
3‐MA: 3‐methyladenine
4‐HNE: 4‐hydroxynonenal
F: respiratory frequency
Fer‐1: ferrostatin‐1
FTH1: ferritin heavy chain 1
GPX4: glutathione peroxidase 4
GSH: glutathione
MDA: malondialdehyde
NCOA4: nuclear receptor coactivator 4
OVA: ovalbumin
PEF: Peak Expiratory Flow
Penh: enhanced pause
PIF: Peak Inspiratory Flow
ROS: reactive oxygen species
SLC7A11: solute carrier family 7 member 11
TV: Tidal Volume
WBP: whole‐body plethysmography

Asthma is one of the most prevalent chronic diseases worldwide, leading to significant healthcare burdens [1, 2]. Asthma exhibits marked heterogeneity in clinical symptoms, severity, and therapeutic responses [3, 4, 5]. Although most asthma patients can be effectively managed with bronchodilators or corticosteroids, long‐term treatment may lead to dependency and adverse effects [6]. Furthermore, a subset of patients with severe asthma remains difficult to control under standard therapeutic regimens [7, 8]. Therefore, understanding the pathogenesis and etiology of asthma is of critical importance, as is exploring targeted treatment strategies to enhance patient outcomes.

Asthma has a complicated pathophysiology. Ferroptosis may play a significant role in the development of asthma, according to increasing evidence [9, 10, 11]. There are several ways that ferroptosis contributes to the pathogenesis of asthma. One important mechanism in the progression of asthma is the accumulation of Fe^2+^ in airway epithelial cells, which is followed by the formation of lipid peroxides and reactive oxygen species (ROS) through the Fenton reaction, which results in cell death. This process may be connected to IL‐17A levels [12, 13]. In house dust mite‐induced allergic asthma, increased production of ROS, depletion of glutathione (GSH), and downregulation of glutathione peroxidase 4 (GPX4) and solute carrier family 7 member 11 (SLC7A11) suggest the occurrence of ferroptosis during this process [14]. The addition of ferrostatin‐1 (Fer‐1) partially reversed the effects of lipid peroxidation, further indicating the potential occurrence of ferroptosis [15]. These findings indicate a close relationship between ferroptosis and asthma. Thus, targeting ferroptosis may represent a novel therapeutic approach for asthma.

Autophagy is a metabolic process that breaks down cellular proteins and organelles to maintain the body in a state of equilibrium [16, 17]. Numerous disorders, including cancer and liver fibrosis, are influenced by autophagy [18, 19, 20]. The latest study has revealed that the downregulation of autophagy is related to the therapeutic effect of CD73 inhibition on liver fibrosis [19]. The connection between ferroptosis and autophagy has been revealed in recent years. According to current studies, TMEM164 can sustain autophagosome production while promoting cell ferroptosis [21]. Ferroptosis in renal tissue may result from OTUD5 autophagy‐induced GPX4 degradation [22]. Ferroautophagy, a distinct form of autophagy, has been shown in numerous investigations to be an essential regulatory mechanism of the ferroptosis process [23]. Excessive ferroautophagy can accelerate ferroptosis because it encourages ferritin breakdown and releases Fe^2+^ [24].

Polydatin is the main active ingredient in *Polygonum cuspidatum*, a plant used in traditional Chinese medicine. It is utilized for its antitumor, lipid‐regulating, and lung‐protective properties [25, 26]. According to recent studies, polydatin can prevent a number of illnesses and mechanically reverse ferroptosis [27, 28]. Nevertheless, it is unknown whether polydatin can reduce asthma symptoms by preventing ferroptosis during an asthma attack. Thus, the primary goal of this research was to investigate the underlying mechanism of the protective effect of polydatin on asthma and to present experimental data indicating that polydatin may target the ferroautophagy–ferroptosis pathway in asthma treatment.

## Materials and methods

### Experimental animals and grouping

Fifty SPF‐grade male SD rats (6 weeks old, weighing between 180 and 200 g) were procured from Beijing Vital River Laboratory Animal Technology Co., Ltd. (Animal Production License No.: SCXK(Jing)2021–0006, Animal Qualification Certificate No.: No. 110011231111163824). The rats were accommodated in a clean‐grade animal facility, maintaining controlled conditions: temperature maintained at 21 ± 1 °C, relative humidity between 30% and 40%, and a light/dark cycle of 12 h each (light from 7:00 to 19:00 and darkness from 19:00 to 7:00). After one week of acclimatization, the rats were randomly distributed into the following groups using a random number method: control group, model group, model + polydatin group (termed as polydatin group), model + 3‐methyladenine (3‐MA) group (termed as 3‐MA group), and model + Fer‐1 group (termed as Fer‐1 group), with each group consisting of 10 rats. The animal protocols were approved by the Experimental Animal Management and Ethics Committee of Hebei University of Chinese Medicine (No. DWLL202312022).

### Model establishment and pharmacological intervention

The relevant literature describes the establishment of an asthma model in rats, encompassing two phases: sensitization and challenge. Throughout the sensitization phase, the model group rats were administered an intraperitoneal injection of 1% ovalbumin (OVA) sensitization solution (1 mL·200 g^−1^, containing 1 mg OVA and 100 mg aluminum hydroxide powder, with physiological saline adjusting the total volume to 1 mL) on Day 1 and again on Day 8. On the other hand, the control group rats received a physiological saline injection of equivalent volume.

During the challenge phase, starting from Day 15, the model group rats were subjected daily to nebulization with a 1% OVA solution (comprising 200 mg OVA and 20 mL of physiological saline). The control group rats underwent nebulization with the same volume of physiological saline. These nebulization procedures were conducted daily for 4 weeks, with each session lasting 30 min. Corresponding drug interventions were administered 2 h before each nebulization challenge, executed once‐daily.

In particular, the control group and the model group received physiological saline (10 mL·kg^−1^·day^−1^) via gavage. The polydatin group was administered a gavage of polydatin extract suspension (200 mg·kg^−1^·day^−1^, dissolved in physiological saline, 10 mL·kg^−1^·day^−1^) through gavage. The 3‐MA group received an intraperitoneal injection of 3‐MA (30 mg·kg^−1^·day^−1^, dissolved in 1% DMSO), and the Fer‐1 group was given an intraperitoneal injection of Fer‐1 (2 mg·kg^−1^·day^−1^, dissolved in 1% DMSO).

### General state observation

We observed the activity status, fur condition, food intake, and body weight of the rats in each group. Records of body weight and food intake were taken on Days 1, 8, 15, 22, 29, 36, and 43. The technique for calculating food intake was as follows: Every group comprised 10 rats that were allotted among three boxes, housing three, three, and four rats, respectively. The total food intake for each box was computed by subtracting the leftover food from the initial food amount. Food intake for every rat was determined by dividing the overall food intake of the box by the number of rats present in the box.

### Behavioral changes

During the final nebulization process, the incubation period of asthma in each group of rats, as well as the number of times the rats scratched their noses and sneezed within the first minute, was observed and recorded. The incubation period of asthma was defined as the time from the inception of the nebulization to the emergence of respiratory distress and other symptoms related to asthma, such as nose scratching and facial rubbing in the rats. This period was monitored and noted in seconds over 6 min; if the latency period exceeded 6 min, it was recorded as 360 s.

### Noninvasive pulmonary function testing

One hour after the last nebulization challenge was completed, each rat was placed in an individual plethysmographic chamber. Three rats were measured in each round. Once the rats were stable, a whole‐body plethysmography (WBP) device was utilized to assess their pulmonary function parameters. It is crucial to maintain a quiet environment during the experiment.

### Sample collection and processing

After the completion of nebulization, all the rat groups underwent a 24‐h fasting period, although they still had free access to water. Blood from the abdominal aorta was taken from anesthetized rats, and the supernatant was retained for immunoglobulin E (IgE) detection. The rats were then euthanized, and their thoracic cavities were quickly opened to fully expose the lungs. These lungs were carefully removed and rinsed with 4% precooled physiological saline. A tissue sample from the left lung, measuring 1.0 × 1.0 × 0.3 cm, was fixed in 4% paraformaldehyde. After 24‐h fixation, the lung tissue was dehydrated, embedded in paraffin, and cut into 4‐μm‐thick sections for pathological staining. The remaining tissue was placed in cryogenic tubes, rapidly frozen in liquid nitrogen, and subsequently stored at −80 °C for further analysis.

### HE staining

The lung tissue was fixed in 4% paraformaldehyde, dehydrated through an alcohol gradient, embedded in paraffin, and sectioned into 4‐μm slices. These slices underwent deparaffinization and sequential incubation in hematoxylin solution for 5 min, followed by differentiation in 1% hydrochloric acid alcohol for 2 s and staining with eosin solution for 1 min. After each step, the specimens were rinsed with tap water for 3 min. After routine dehydration and mounting, the samples were observed under an optical microscope (DM6B; Leica, Wetzlar, Germany).

### Masson staining

After the process of deparaffinization, the sections were incubated in a hematoxylin staining solution for 5–10 min. The sections were then thoroughly rinsed with running water, treated using hydrochloric acid ethanol, and reverted to a blue color. After this, the sections were incubated in Masson's acid fuchsin solution for 5 min. A rapid rinse using acetic acid solution was carried out, followed by staining in a 1% phosphomolybdic acid solution for between 3 and 5 min. Subsequently, the sections were directly stained with aniline blue solution for roughly 5 min, washed again with the same acetic acid solution, and then went through the typical process of dehydration and mounting. In the final phase, the tissues were examined under an optical microscope (DM6B; Leica, Wetzlar, Germany).

### PAS staining

After deparaffinization, the sections were incubated in a solution of periodic acid for 10 min. They were then rinsed with water for an additional 10 min, followed by a 10‐min incubation in Schiff's reagent. The sections were rinsed again, this time for 5 min, then stained with hematoxylin for 3 min, and rinsed once more for 5 min. Finally, the sections were routinely dehydrated and mounted. Observations were conducted using an optical microscope (DM6B; Leica, Wetzlar, Germany).

### Serum IgE detection

After collecting blood from the abdominal aorta, allow it to stand at room temperature for 30 min. Then, centrifuge (1000 × ***g***, 20 min, 4 °C) and obtain the supernatant. The serum IgE levels were measured using the Rat IgE ELISA Kit (D731065; Sangon Biotech, Shanghai, China). Add 100 μL of the standard substance to each sample and incubate at 37 °C. After 90 min, incorporate 100 μL of biotinylated antibody working solution for further incubation at 37 °C for 60 min. Following five wash cycles, introduce 100 μL of HRP‐labeled streptavidin working solution, and then incubate for an additional 30 min. Rinse the plate five times and apply 60 μL of color reagent, then incubate the plate for another 15 min at 37 °C. Read the optical density (OD) value at 450 nm after adding 50 μL of termination solution.

### Immunohistochemistry

Lung tissue sections were subjected to hydration. Antigen repair was performed, followed by three washes with PBST, each lasting for 1 min. The sections were then incubated with the nuclear receptor coactivator 4 (NCOA4) primary antibody (1 : 50, A5695; ABclonal, Wuhan, China) and the ferritin heavy chain 1 (FTH1) primary antibody (1 : 200, 4393; Cell Signaling Technology, Massachusetts, USA) at 25 °C for 1.5 h, followed by a rinse using PBS (three 5‐min washes). Subsequently, the sections were incubated with the secondary antibody, goat anti‐rabbit IgG labeled with horseradish peroxidase (1 : 100, PTM‐6261; PTM BIO, Hangzhou, China), at 25°C for 30 min. This was followed by another rinse using PBS (three 5‐min washes). The next step was processing with DAB at 25°C for 8 min, counterstaining with hematoxylin for 3 min, and soaking in an EDTA repair solution for 2 min. The sections were then observed under an optical microscope after being dehydrated, rendered transparent, and sealed.

### Co‐immunoprecipitation (co‐IP)

A suitable amount of lung tissue was taken, washed three times with precooled PBS, and then cut into small pieces before being placed in a homogenization tube. Protease inhibitors and IP lysis buffer (10 times the tissue volume) were subsequently added for homogenization. The homogenate was then transferred to a 1.5‐mL centrifuge tube and lysed on ice for 30 min. After lysing, the mixture was centrifuged at 12 000 ***g*** for 10 min at 4 °C. The supernatant was collected to determine protein concentration using the BCA method. The negative control (IgG) group and the experimental group had 1.0 μg IgG + 20 μL protein A/G beads added to the protein supernatant, while only 20 μL of protein A/G beads was added to the negative control group. The samples were then incubated at 4 °C for 1 h. After incubation, the samples were centrifuged at 22 000 ***g*** for 5 min at 4 °C, and the supernatant was collected. Primary antibodies NCOA4 (1 : 30, ab314553; ABcam, Cambridge, UK) or FTH1 (1 : 50, 4393; Cell Signaling Technology, USA) were added to the samples, which were incubated overnight at 4 °C. Following this incubation, 80 μL of protein A/G beads were added to the mixture and incubated for another 2 h at 4 °C. The samples were then centrifuged at 2000 ***g*** for 5 min at 4 °C, and the supernatant was discarded. The immunoprecipitated complexes were then collected and washed four times with 1 mL of precooled IP lysis buffer. After each wash, centrifugation was carried out (2000 ***g*** for 5 min at 4 °C) and the supernatant was discarded each time. Lastly, 80 μL of 1× reducing sample buffer was added, and the samples were boiled for 10 min. The supernatant was collected after centrifugation at 1000 ***g*** for 5 min at 4 °C. After SDS/PAGE electrophoresis and membrane transfer, the primary antibody NCOA4 (1 : 1000) and FTH1 (1 : 1000) were incubated overnight. Afterwards, the secondary antibody HRP‐conjugated Goat Anti‐Rabbit IgG (1 : 5000, GB23303; Servicebio, Wuhan, China) was added and incubated for 1 h, followed by luminescence imaging.

### Quantitative real‐time polymerase chain reaction (qRT‐PCR)

Appropriate amount of lung tissue was taken, RNA lysate was added and homogenized with an electric homogenizer. Total RNA was extracted using the Eastep Super Total RNA Extraction Kit (LS1040; Promega, Madison, USA). cDNA was synthesized by GoScript Reverse Transcription System (A5000; Promega, Madison, USA). Real‐time quantitative fluorescent PCR (CFX96; BIO‐RAD, Shanghai, China) was used for amplification under the following conditions: predenaturation at 95 °C for 10 min, denaturation at 95 °C for 15 s, and annealing at 60 °C for 1 min, repeated for 40 cycles. With β‐actin as the control, the relative expression of the target gene was analyzed by 2^−ΔΔ*C*t^. Primers for NCOA4, FTH1, Bcl‐2 homologous domain protein (Beclin1), ubiquitin‐binding protein p62 (P62), GPX4, SLC7A11 are shown in Table 1.

**Table 1:** Primers for NCOA4, FTH1, Beclin1, P62, GPX4, SLC7A11 and β‐actin.

Gene	Primer sequence (5′–3′)	Length (bp)
NCOA4	Forward: GTTCACGCAGCAGTTTTCGT Reverse: CTCCTTGCATCACTGCACCT	127
FTH1	Forward: CCTTTGCAACTTCGTCGCTC Reverse: TCCGAGTCCTGGTGGTAGTT	115
Beclin1	Forward: GAATGGAGGGGTCTAAGGCG Reverse: TCGTGTCCAGTTTCAGAGGC	87
P62	Forward: TGCTCCATCAGAGGATCCCA Reverse: TTTCTGCAGAGGTGGGTGTC	146
GPX4	Forward: AAAGTCCTAGGAAGCGCCCA Reverse: GGGTTGAAAGGCTCGGGAAT	106
SLC7A11	Forward: AACCCAAGTGGTTCAGACGATT Reverse: GGCAGATGGCCAAGGATTTGA	123
β‐Actin	Forward: GCAGGAGTACGATGAGTCCG Reverse: ACGCAGCTCAGTAACAGTCC	74

### Western bolt

Lung tissue from rats was minced and homogenized in lysis buffer. The homogenate was centrifuged, and the supernatant was collected. We measured the protein concentration using a BCA kit (PC0020; Solarbio, Beijing, China) and a spectrophotometer. SDS/PAGE gels were prepared and placed in an electrophoresis chamber filled with an electrophoresis buffer. We loaded the same amounts of samples into the wells for electrophoresis (80 V for 40 min. After the marker had migrated, we adjusted the voltage to 120 V for 50 min). Once the bromophenol blue had reached the bottom of the gel, the proteins were transferred to a nitrocellulose (PVDF) membrane (100 mA for 80 min). After the transfer, we coated the membrane with 5% skim milk powder for 2 h and rinsed it. The membrane received primary antibodies such as NCOA4 (1 : 1000, A5695; ABclonal, Wuhan, China), FTH1 (1 : 1000, 4393; Cell Signaling Technology, Massachusetts, USA), Beclin1 (1 : 1000, A21695; ABclonal, Wuhan, China), SLC7A11 (1 : 1000, A25302; ABclonal, Wuhan, China), GPX4 (1 : 1000, 30 388‐1‐AP; Proteintech, Illinois, USA), and P62 (1 : 4000, NBP1‐48320; Novus Biologicals, Colorado, USA) for wave incubation at 4 °C overnight, on top of the bed. The following day, we washed the membrane with TBST (3 times, 10 min per time). The membrane was then incubated with Goat anti‐rabbit IgG labeled with horseradish peroxidase (1 : 10 000, PTM‐6261; PTM BIO, Hangzhou, China) at room temperature for 1 h. Afterward, the membrane was rinsed with TBST (3 times, 10 min per time) and PBS once for 5 min. Finally, we visualized the bands using an ECL reagent in a gel imaging system.

### Prussian blue staining

The sections were deparaffinized and then immersed in Prussian blue staining solution for 30 min. This was followed by a water rinse before staining the sections with a nuclear fast red solution for 5 min. After another water rinse, the samples underwent routine dehydration and were mounted with neutral resin. Upon completion of the staining process, the tissues were examined using an optical microscope (DM6B; Leica, Wetzlar, Germany).

### Detection of Fe^2+^, GSH, MDA, and 4‐HNE in lung tissue

The lung tissue was meticulously rinsed with cold PBS and then homogenized in a buffer on ice, a process subsequently followed by centrifugation. The supernatant was then acquired for use in the next set of experiments. Fe^2+^ (A039‐2‐1; Nanjing Institute of Bioengineering, China), GSH (A006‐2‐1; Nanjing Institute of Bioengineering, China), malondialdehyde (MDA) (A003‐1‐2; Nanjing Institute of Bioengineering, Nanjing, China), and 4‐hydroxynonenal (4‐HNE) (D751041; Shenggong Biological Engineering Co., LTD. BBI, Shanghai, China) levels within the lung tissue were gauged following the guidelines provided by their respective assay kits.

### Statistics

The data were statistically analyzed using the SPSS 23.0 software. If the data conformed to a normal distribution, the measurements were expressed as mean ± standard deviation. One‐way analysis of variance (ANOVA) served to compare data between multiple groups. Pairwise comparisons were made using the SNK‐q method for homogeneous variances and Dunnett's T3 test for heterogeneous variances. A *P*‐value less than 0.05 was deemed statistically significant.

## Results

### General state and behavioral changes

We observed and recorded factors such as fur quality, activity levels, food intake, and body weight in each group of rats. The results indicated that the rats in the control group displayed good mental states—they were agile and active, had shiny, smooth fur, and their food intake and body weight were normal.

**Fig. 1:**
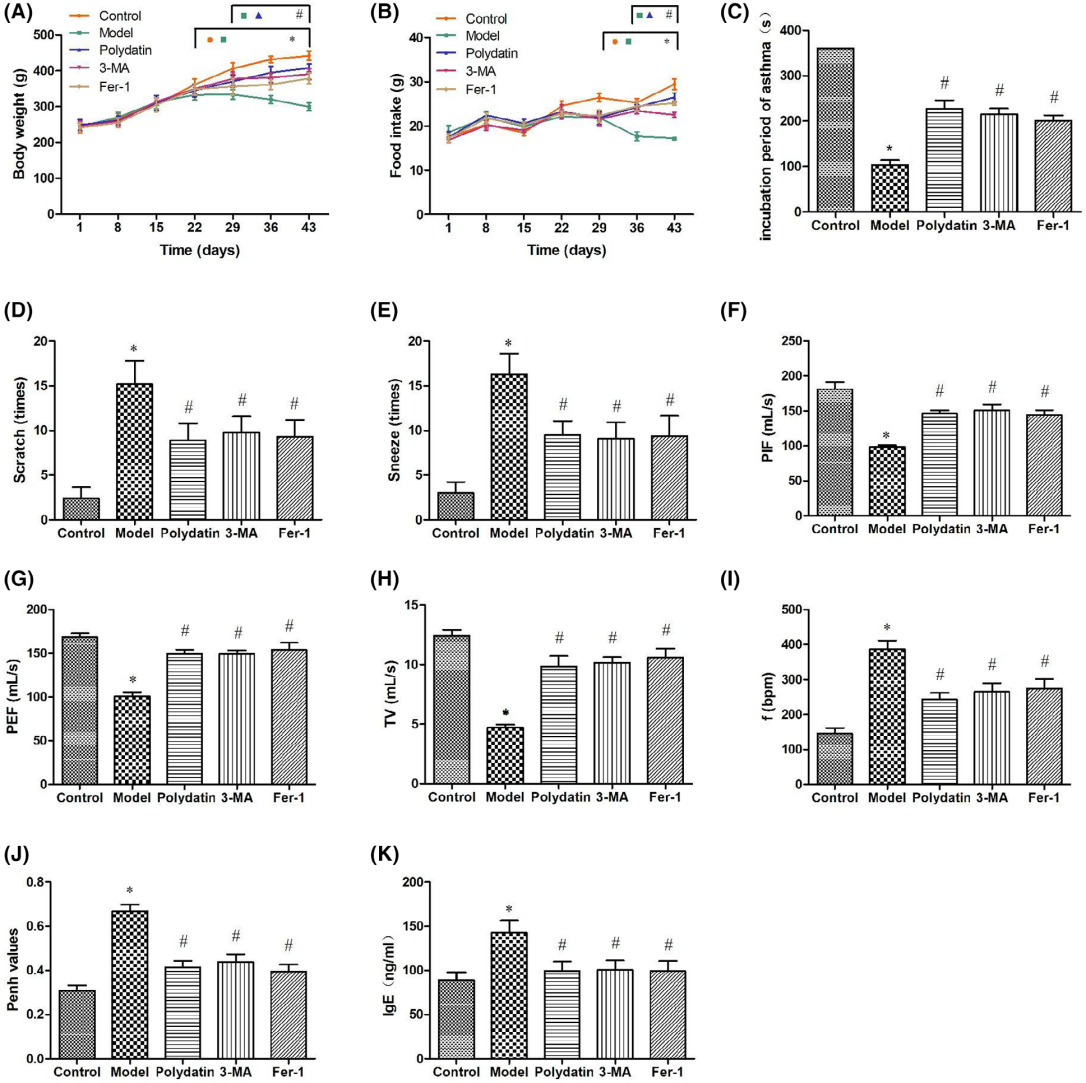
General status, behavioral alterations, lung function, and serum IgE levels in asthmatic rats. (A, B) Changes in body weight (*n* = 10) and food intake (*n* = 3) among different groups of rats. (C–E) Variations in the incubation period of asthma, times of nose scratch and sneeze among different groups of rats (*n* = 10). (F–J) Comparison of peak inspiratory flow (PIF), peak expiratory flow (PEF), tidal volume (TV), respiratory frequency (f) and enhanced pause (Penh) among different groups of rats (*n* = 10). (K) Serum IgE levels among different groups of rats (*n* = 10). * indicates compared to the control group, *P* < 0.05; # indicates compared to the model group, *P* < 0.05. Data were presented as mean ± SD. The statistical testing methods used are SNK‐q or Dunnett's T3.

In contrast, the rats in the model group exhibited signs of agitation at the beginning of nebulization. As the duration of nebulization increased, these rats displayed lethargy and a tendency to curl up. Their fur became erect and lost its shine. Persistent symptoms such as dyspnea, wheezing, and nodding movements became apparent over time. The food intake and body weight of these model group rats gradually increased at a slow rate until an eventual decline occurred, with a significant reduction in food intake starting from Day 29 post modeling (*P* < 0.05). Additionally, there was a noticeable decrement in body weight beginning on Day 22 (*P* < 0.05) compared to the control group. An intervention with polydatin extract yielded improvement in the rats' mental state and fur quality. Furthermore, their food intake increased starting from Day 36 (*P* < 0.05), and their weight escalated from Day 29 (*P* < 0.05) when compared to the model group (Fig. 1A,B).

Compared with the control group, the model group rats had a shortened incubation period of asthma (*P* < 0.05) and displayed an increase in instances of nose scratching and sneezing (*P* < 0.05). However, the polydatin, 3‐MA, and Fer‐1 groups exhibited a prolonged incubation period of asthma (*P* < 0.05) and a decrease in instances of nose scratching and sneezing (*P* < 0.05) (Fig. 1C–E).

### Noninvasive pulmonary function and serum IgE levels

The WBP system was used to evaluate pulmonary function parameters. In comparison with the control group, the model group rats displayed a decrease in peak inspiratory flow (PIF), peak expiratory flow (PEF), and tidal volume (TV) (*P* < 0.05), as well as increased respiratory frequency (f) and enhanced pause (Penh) (*P* < 0.05). When compared to the model group, the polydatin, 3‐MA, and Fer‐1 groups exhibited an increase in PIF, PEF, and TV (*P* < 0.05), in conjunction with a decrease in f and Penh (*P* < 0.05). This indicates that these treatments significantly improved pulmonary function in asthmatic rats (Fig. 1F–J). Compared with the control group, the model group displayed elevated serum IgE levels (*P* < 0.05); nonetheless, the polydatin, 3‐MA, and Fer‐1 groups showed a reduction in serum IgE levels in comparison with the model group (*P* < 0.05) (Fig. 1K).

### Pathological morphological changes

**Fig. 2:**
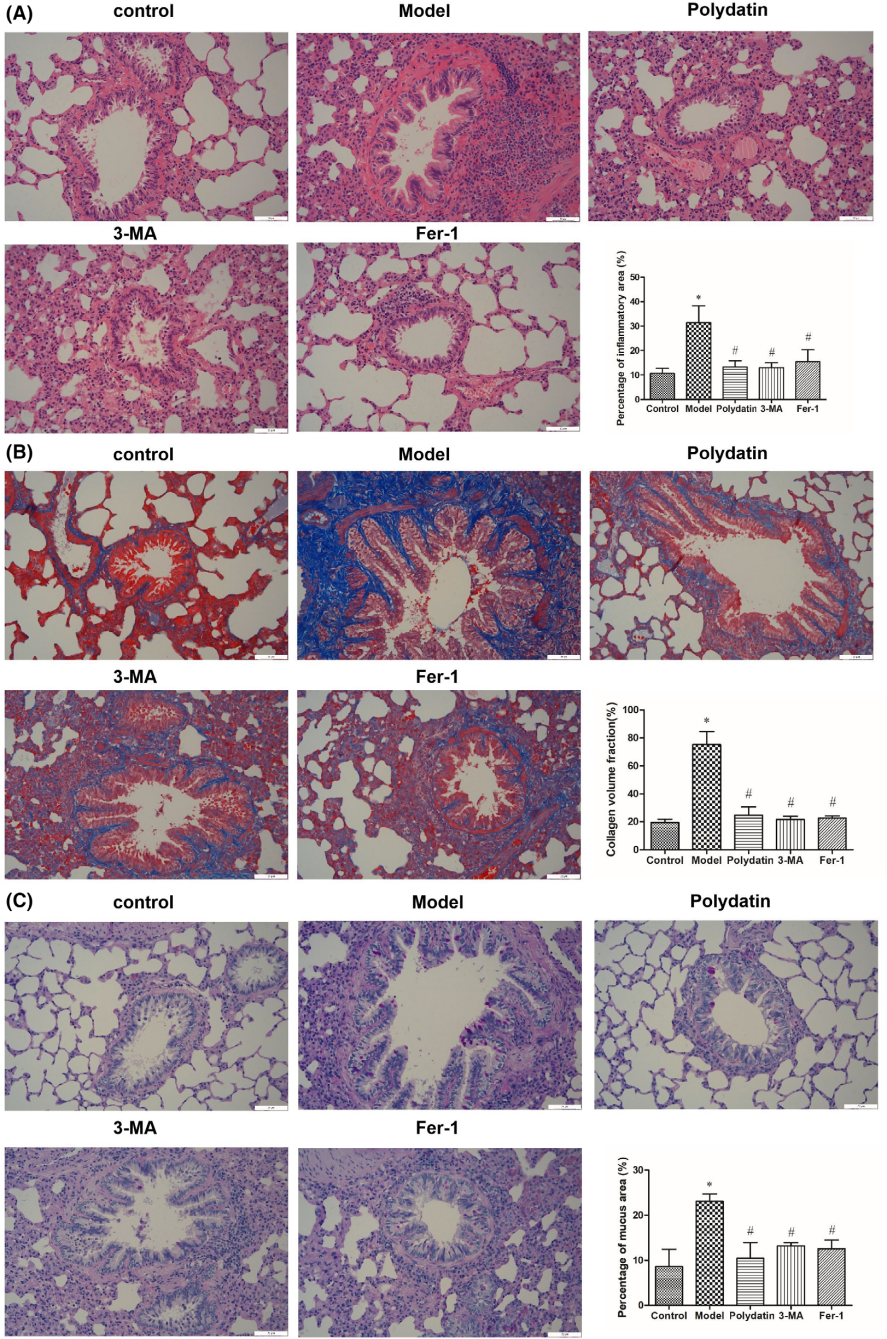
Effects of polydatin on lung histology in asthmatic rats. (A) Hematoxylin–eosin staining (*n* = 3). (B) Masson staining (*n* = 3). (C) Periodic acid‐Schiff staining (*n* = 3). Scale bar = 50 μm. * indicates comparison to the control group, *P* < 0.05; # indicates comparison to the model group, *P* < 0.05. Data were presented as mean ± SD. The statistical testing method used is SNK‐q.

HE staining revealed that the tracheal wall structure of the rats in the control group was normal; there was no aggregation of inflammatory cells observed. In contrast, rats in the model group displayed substantial infiltration of inflammatory cells. When compared with the model group, the polydatin, 3‐MA, and Fer‐1 groups displayed a reduction in the number of inflammatory cells around the trachea (*P* < 0.05) (Fig. 2A). Masson staining showed that the rats in the model group had an evident buildup of collagen fibers around the airways compared to the control group. The treatments with polydatin, 3‐MA, and Fer‐1 markedly decreased the deposition of collagen fibers around the airways when compared with the model group (*P* < 0.05) (Fig. 2B). PAS staining showed that the model group rats demonstrated goblet cell hyperplasia, plus increased secretion of mucus in the airways compared to the control group. The treatments with polydatin, 3‐MA, and Fer‐1 effectively suppressed goblet cell hyperplasia and improved the excessive mucus secretion in the airway epithelium in comparison with the model group (*P* < 0.05) (Fig. 2C).

### Immunohistochemical and co‐IP of NCOA4 and FTH1

**Fig. 3:**
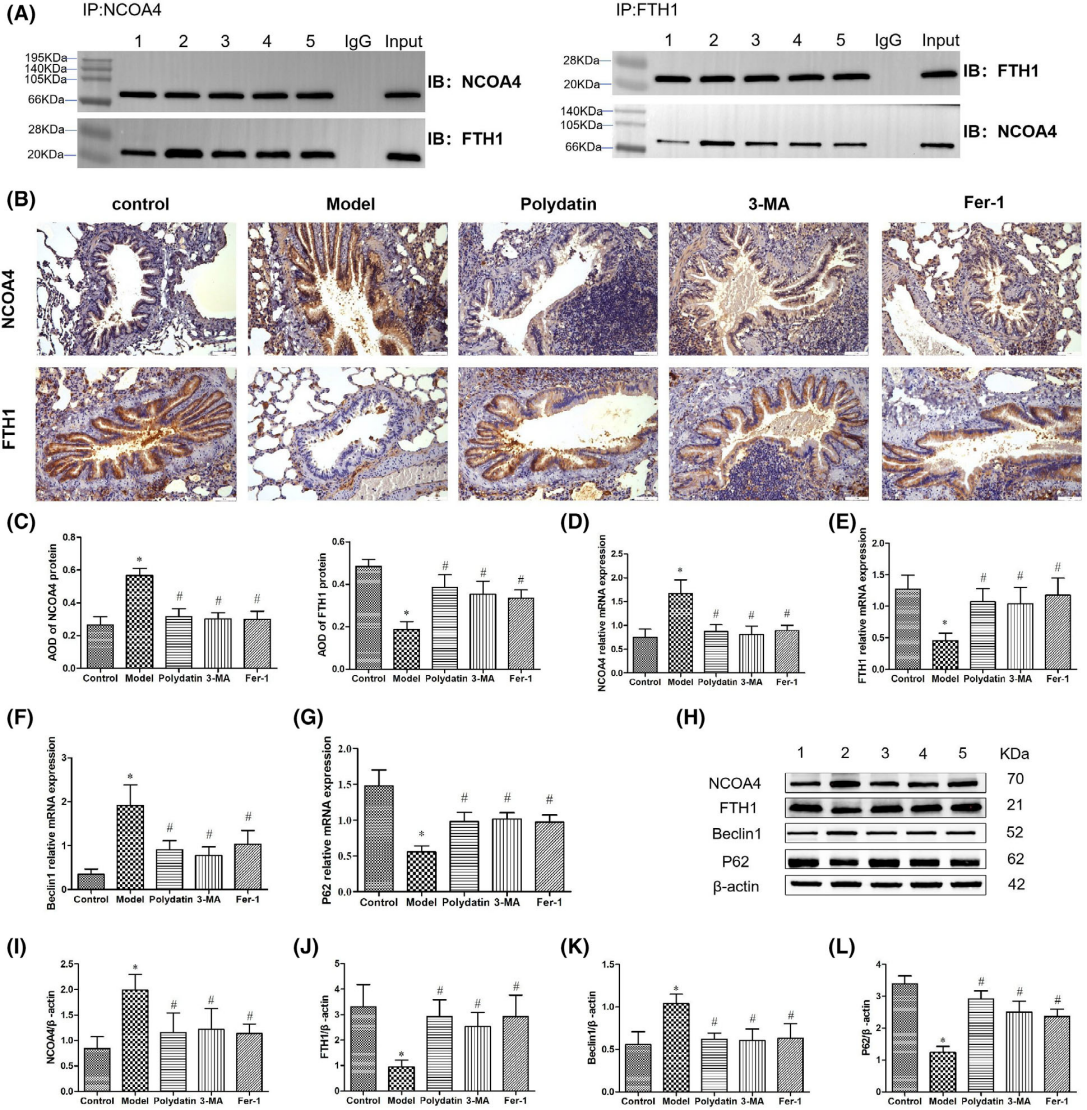
Effects of polydatin on the levels of ferroautophagy‐related indicators in asthmatic rats. (A) Interaction between NCOA4 and FTH1 in rats between groups. (B, C) The expression of NCOA4 and FTH1 in lung tissue of rats in each group (*n* = 6). Scale bar = 50 μm. (D–G) The mRNA expression of NCOA4, FTH1, Beclin1, and P62 in lung tissues of rats among different groups (*n* = 10). (H) Western blotting bands of NCOA4, FTH1, Beclin1, and P62 in lung tissue of rats among different groups. (I–L) The protein expression of NCOA4, FTH1, Beclin1, and P62 in lung tissues of rats among different groups (*n* = 3). 1, control group; 2, model group; 3, polydatin group; 4, 3‐MA group; 5, Fer‐1 group; * indicates compared to the control group, *P* < 0.05; # indicates compared to the model group, *P* < 0.05. Data were presented as mean ± SD. The statistical testing methods used are SNK‐q or Dunnett's T3.

The interaction between NCOA4 and FTH1 in the lung tissues of the model group of rats was more enhanced than in the control group; however, after administering pharmacological treatments (such as polydatin, 3‐MA, and Fer‐1), this interaction was lessened (Fig. 3A). The NCOA4 expression in airway epithelial cells was higher in the model group than in the control group, while the FTH1 expression was lower. In contrast, compared with the model group, the polydatin group demonstrated a decrease in NCOA4 expression and an increase in FTH1 expression in airway epithelial cells (Fig. 3B,C).

### Ferroautophagy‐related genes mRNA and protein expression

In comparison with the control group, the model group rats displayed elevated mRNA expression levels of NCOA4 and Beclin1 within lung tissues (*P* < 0.05), whereas FTH1 and P62 expression levels were reduced (*P* < 0.05). Compared with the model group, the polydatin, 3‐MA, and Fer‐1 groups showed decreased mRNA levels of NCOA4 and Beclin1 in lung tissues (*P* < 0.05), along with increased FTH1 and P62 expression levels (*P* < 0.05) (Fig. 3D–G). Model group rats exhibited a higher degree of NCOA4 and Beclin1 protein expression within their lung tissues compared to the control group (*P* < 0.05), but lower protein expression of FTH1 and P62 (*P* < 0.05). Contrary to the model group, the polydatin, 3‐MA, and Fer‐1 groups displayed diminished NCOA4 and Beclin1 protein expression (*P* < 0.05) and elevated FTH1 and P62 protein expression (*P* < 0.05) (Fig. 3H–L).

### Fe^3+^ deposition in airways and surrounding tissues

**Fig. 4:**
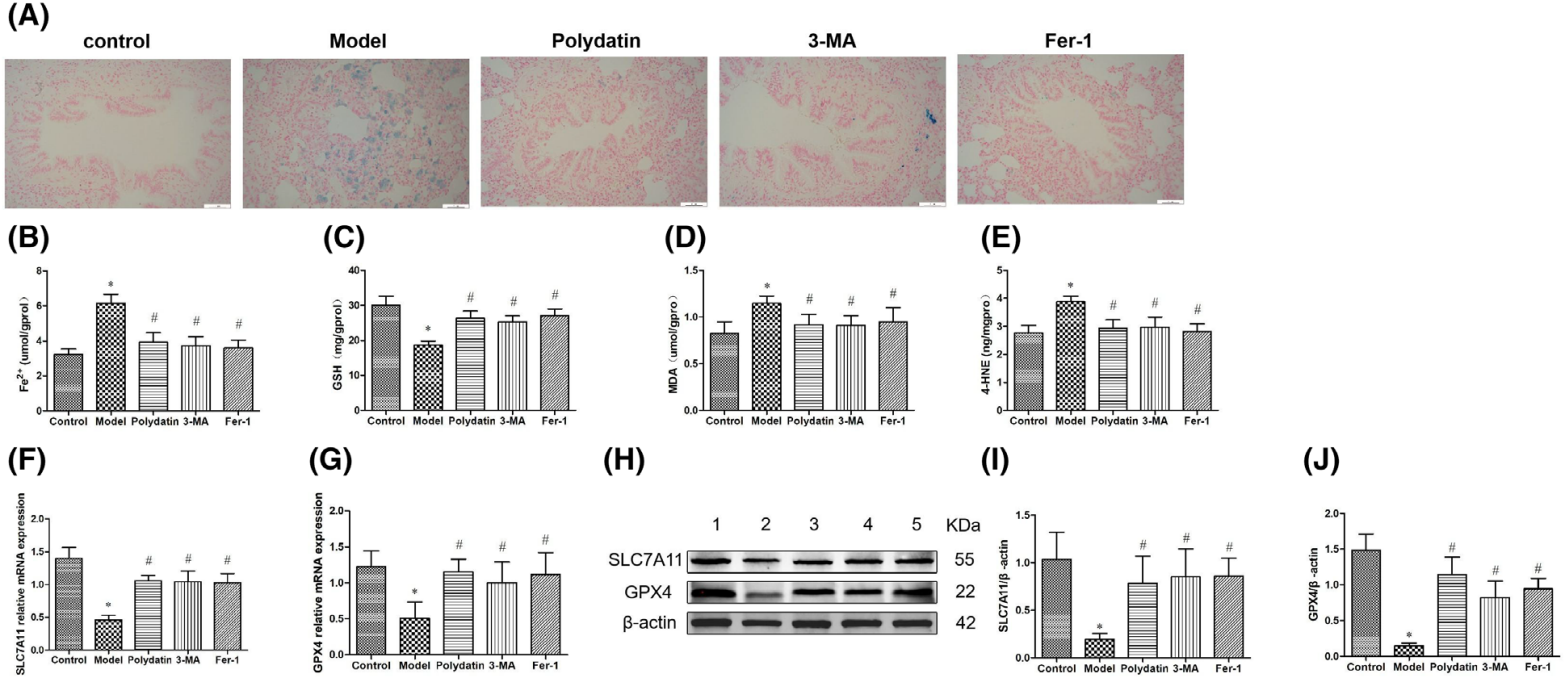
Effect of polydatin on the levels of ferroptosis‐related indicators in asthmatic rats. (A) Fe^3+^ deposition in lung tissue of rats in each group. Scale bar = 50 μm. (B) Fe^2+^ levels in lung tissue of rats in each group (*n* = 10). (C–E) Levels of GSH, MDA, and 4‐HNE in lung tissues of rats in each group (*n* = 10). (F, G) The mRNA expression of SLC7A11 and GPX4 in lung tissues of rats among different groups (*n* = 10). (H) Western blotting bands of SLC7A11 and GPX4 in lung tissue of rats among different groups. (I, J) The protein expression of SLC7A11 and GPX4 in lung tissues of rats among different groups (*n* = 3). 1, control group; 2, model group; 3, polydatin group; 4, 3‐MA group; 5, Fer‐1 group; * indicates compared to the control group, *P* < 0.05; # indicates compared to the model group, *P* < 0.05. Data were presented as mean ± SD. The statistical testing method used is SNK‐q.

Rats in the model group exhibited higher levels of Fe^3+^ deposition in airways and their surrounding tissues compared to the control group rats. However, the polydatin, 3‐MA, and Fer‐1 groups displayed less Fe^3+^ deposition in these areas compared to the model group (*P* < 0.05) (Fig. 4A).

### Fe^2+^, MDA, 4‐HNE content, and GPX4, SLC7A11 mRNA and protein expression

Compared to the control group, the rats in the model group demonstrated elevated expression levels of Fe^2+^, MDA, and 4‐HNE in lung tissues (*P* < 0.05). Conversely, GSH levels decreased (*P* < 0.05) (Fig. 4B–E). Furthermore, the expression levels of GPX4 and SLC7A11 mRNA and protein diminished (*P* < 0.05) (Fig. 4F–J). When compared to the model group, the groups treated with polydatin, 3‐MA, and Fer‐1 showed decreased expression levels of Fe^2+^, MDA, and 4‐HNE in lung tissues (*P* < 0.05). These groups also exhibited an increase in GSH levels (*P* < 0.05), as well as an upsurge in the expression of GPX4 and SLC7A11 mRNA and protein (*P* < 0.05).

## Discussion

### Main interpretation

This study succeeded in establishing an asthma rat model via intraperitoneal injection and aerosolized administration of an OVA solution. When individuals with asthma encounter allergens in the respiratory tract, their immune system generates a considerable amount of IgE. This IgE then binds with respiratory tract mast cells and eosinophils. This binding action prompts these cells to release a substantial amount of histamine and other inflammatory mediators, causing airway inflammation and constriction, which characterize the symptoms of asthma. We confirmed the successful creation of our asthma model by observing the overall condition and behavioral changes of the rats, changes in pulmonary function, serum IgE levels, and pathological observations. Additionally, we demonstrated that polydatin, 3‐MA, and Fer‐1 could lessen asthma symptoms, mitigate airway inflammatory responses, improve pulmonary function, and lower serum IgE levels.

Most previous studies on the mechanisms of asthma‐induced injury have focused primarily on the excessive release of ROS, calcium overload, inflammatory responses, and apoptosis [3, 4]. In recent years, ferroptosis has attracted increasing interest, and studies have shown that disruptions in iron metabolism, lipid peroxidation, and amino acid metabolism lead to ferroptosis in airway epithelial cells. This process results in the generation of excessive ROS, which in turn activate immune inflammatory responses and exacerbate the clinical symptoms of asthma patients [9, 10, 11]. Ferroptosis may be a key factor in the pathological process of asthma. Moreover, several regulatory factors associated with ferroptosis, such as Nrf2, heme oxygenase‐1, and ferroptosis suppressor protein 1, have also been implicated in the pathogenesis of asthma. Studies suggest that ferroptosis inhibitors may alleviate asthma symptoms, indicating that inhibiting ferroptosis could be a potential therapeutic approach for managing asthma [9, 10, 11].

Ferroptosis is characterized by the Fe^2+^ content, the accumulation of lipid peroxides, and a deficiency of GPX4 [29]. Lipid peroxidation, a crucial aspect of ferroptosis, can be regulated by lipoxygenases (LOXs), which catalyze MDA and 4‐HNE, promoting the occurrence of ferroptosis [30]. GPX4 functions as a negative regulator of ferroptosis by converting lipid hydroperoxides into lipid alcohols [31, 32]. SLC7A11, which promotes the absorption of cysteine into cells and its reduction into cysteine for GSH synthesis, is another significant signal for ferroptosis and protects cells from oxidative damage [33, 34, 35]. Fe^2+^, MDA, 4‐HNE, GPX4, and SLC7A11 were therefore utilized in this study as ferroptosis markers. Our findings confirmed that ferroptosis increased in the model group, supporting the theory that ferroptosis plays a role in the development of asthma. The deposition of Fe^3+^ around the airways and the alterations in these markers formed the basis for these changes. We found that the effects of indicators could be effectively reversed and that symptoms of asthma could be reduced by polydatin, 3‐MA, and Fer‐1. Our results demonstrated that polydatin could prevent ferroptosis from occurring during asthma treatment.

Another primary subject of this study was polydatin. It is derived from the traditional Chinese herb *Polygonum cuspidatum* and is the primary active compound within this plant. Studies have shown that polydatin is involved in the regulation of autophagy in conditions such as nonalcoholic liver injury and myocardial ischemia. Autophagy is a highly conserved catabolic method that degrades proteins and organelles through lysosomal pathways [36, 37, 38]. Some reports suggest that activated autophagy may be associated with asthma pathogenesis [39, 40, 41]. However, it is still unclear whether polydatin plays a role in the regulation of autophagy during asthma.

Beclin‐1 can form a complex with other proteins to initiate the autophagy process. The absence of Beclin‐1 leads to autophagy inhibition, resulting in the dysregulation of cellular autophagy and an increased risk for various diseases such as neurodegenerative disorders, cancer, and infections [42, 43]. During the formation of autophagosomes, P62 acts as a bridge that selectively encapsulates the connection between LC3 and polyubiquitinated proteins and is subsequently degraded by proteases within the autophagolysosome [44, 45]. The expression level of P62 is negatively associated with autophagic activity [44, 45]. Both Beclin‐1 and P62 are extensively used as autophagy markers in research. In the experimental group, Beclin‐1 expression was elevated, whereas P62 expression was decreased. These changes were inhibited upon intervention with polydatin. These results suggest that autophagy is amplified in asthma and that polydatin can suppress this phenomenon.

Ferroautophagy is a selective autophagic process that primarily involves ferritin degradation. This process plays a crucial role in various biological functions, including intracellular iron metabolism, oxygen transport, redox reactions, and metabolite synthesis. Moderate ferroautophagy is essential for maintaining stable intracellular iron levels. However, excessive ferroautophagy can result in the release of large amounts of free iron, leading to increased generation of ROS and subsequent cellular damage, such as lipid peroxidation. Therefore, ferroautophagy is important for maintaining physiological ferroautophagy in cells. Investigating the regulatory mechanisms and functions of ferroautophagy can help elucidate the pathogenesis of related diseases and reveal new therapeutic targets.

During ferroautophagy, ferritin stores intracellular Fe^3+^, whereas NCOA4 specifically targets FTH1 to autophagosomes, leading to the degradation of ferritin and the release of free Fe^2+^. Under physiological conditions, ferroautophagy maintains the balance of intracellular Fe^2+^. When ferroautophagy is excessively activated, the accumulation of excessive intracellular Fe^2+^ induces GSH depletion and reduces GPX4 expression, resulting in the collapse and rupture of the membrane structure, ultimately leading to cellular ferroptosis. Our study revealed that the model group presented elevated levels of NCOA4 and Beclin1 in lung tissue, whereas FTH1 and P62 expression were downregulated, suggesting that NCOA4‐mediated ferroautophagy is activated during asthma. After polydatin treatment, NCOA4 and Beclin1 levels in rat lung tissue decreased, whereas FTH1 and P62 expression increased, indicating ferroautophagy inhibition.

In our study, polydatin had a similar inhibitory effect on NCOA4‐mediated ferroptosis. We further investigated NCOA4 and FTH1 expression in the presence of polydatin through immunohistochemistry, as well as their interaction through co‐IP experiments. The model group presented increased NCOA4 expression and decreased FTH1 expression in airway epithelial cells. Conversely, in the polydatin group, NCOA4 expression was reduced, and FTH1 expression was increased compared with that in the model group. Additionally, the interaction between NCOA4 and FTH1 was greater in the lung tissue of model group rats than in that of control group rats, whereas, following drug intervention, the interaction between NCOA4 and FTH1 weakened.

We speculate that polydatin influences its interaction with FTH1 by directly binding to NCOA4, a selective transport receptor. Studies have demonstrated that binding NCOA4^383–522^ can successfully prevent the connection between NCOA4 and FTH1, since this amino acid sequence serves as the binding site between NCOA4 and FTH1 [46, 47]. Thus, it is worthwhile to investigate whether polydatin binds to NCOA4^383–522^ and prevents FTH1 from interacting with it.

On the basis of the aforementioned results, our research confirms that ferroptosis, which is mediated by ferroautophagy, is a crucial element in the pathogenic process of asthma. Moreover, polydatin is a significant therapeutic agent in alleviating asthma symptoms and slowing the disease's pathological progression. These findings provide a foundation for the development of new drugs aimed at enhancing asthma treatment. A key contribution of this study is to clarify that polydatin can reduce NCOA4 expression, elevate FTH1 expression, weaken the interaction between NCOA4 and FTH1, and inhibit the ferroptosis–macroautophagy pathway. This contributes to our understanding of the mechanisms by which polydatin improves asthma.

### Limitations

The pathogenic process of human asthma can be replicated in animal models, but these models cannot accurately capture all the features of human asthma. Consequently, additional clinical research is necessary to validate the efficacy of polydatin intervention in asthma. Furthermore, ferroptosis, a recently identified significant pathway impacting the pathogenesis of asthma, warrants further research to determine its regulatory mechanism. Other important mechanisms could potentially affect ferroptosis in asthma. Moreover, as ferroptosis represents a new method of programmed cell death, and given that cell experiments can support these findings, the upcoming research team will conduct relevant cell studies.

### Conclusion

Polydatin can reduce the overload of intracellular Fe^2+^ by inhibiting NCOA4‐mediated ferroautophagy. This results in the inhibition of ferroptosis in the lung tissue of asthmatic rats, thereby alleviating asthma symptoms.

## Conflict of interest

The authors declare no conflict of interest.

## Peer review

The peer review history for this article is available at https://www.webofscience.com/api/gateway/wos/peer‐review/10.1002/2211‐5463.70090.

## Author contributions

WL designing experiments and writing the manuscript. YWT and WKL raising rats, modeling, and testing indicators. FF and JPW organizing data, drawing tables, and graphics. CYF supervising the experimental process and revising the manuscript. All authors have read and approved the final version of the manuscript.

## Data Availability

The original data supporting this study will be provided by the authors upon request without reservation.
